# Five Fruit and Vegetable a Day Does Not Reflect the Upward Trend of Obesity in the U.S.

**DOI:** 10.23937/2572-3278.1510038

**Published:** 2019-07-27

**Authors:** Yilin Yoshida, Chester L Schmaltz, Jeannette Jackson-Thompson, Adam Bouras, Esmaeel Rahmani, Eduardo J Simoes

**Affiliations:** 1 Department of Medicine, Section of Endocrinology, School of Medicine, Tulane University, USA; 2 Department of Health Management and Informatics, School of Medicine, University of Missouri-Columbia, Columbia, USA; 3 Missouri Cancer Registry and Research Center, University of Missouri-Columbia, Columbia, USA; 4 Griffin Hospital, USA

**Keywords:** 5 fruit and vegetable a day, Obesity, Trends, BRFSS

## Abstract

**Objective:**

After almost three decades of U.S. surveillance in fruit and vegetable (F&V) intake and obesity, it is important to evaluate their usefulness for monitoring prevention and health promotion efforts in public health. We used U.S. surveillance data to evaluate whether the 16-year trends of F&V intake, measured by the prevalence of eating five or more servings of fruits and vegetables a day (FV5/day) is related to obesity trend as measured by its prevalence in the same period. We also evaluated whether trends in the prevalence of FV5/day by important sociodemographic factors (age, race/ethnicity, etc.) could explain the findings.

**Study design:**

A secondary analysis of U.S. adults (≥ 18 years) from the Behavioral Risk Factor Surveillance System (BRFSS) (1994–2009).

**Methods:**

We categorized survey subjects for their F&V intake derived from the BRFSS six-question food frequency questionnaire into two groups: < FV5/day vs. ≥ FV5/day. Obesity was defined as BMI ≥ 30. We used logistic regressions to compute predicted prevalence of FV5/day and obesity, and to estimate the odds ratio of FV5/day by obesity and levels of sociodemographic, stratified by year.

**Results:**

Between 1994 and 2009, the prevalence of FV5/day hovered around 25% among U.S. adults, while the obesity prevalence steadily increased from 14.8% to 27.4%. As measured through odds ratio, an inverse association between FV5/day and obesity was only observed in 55+, but not in other age, racial/ethnic or education groups.

**Conclusions:**

Between 1994 and 2009, we could not confirm a decrease in the prevalence of FV5/day associated with an increase in obesity prevalence, except for age 55+ group. Known disparities in FV5/day and obesity across sociodemographic factors persisted over the study period. FV5/day may be an inappropriate measure of total calories derived from eating fruits and vegetables. Its use to measure impact of public health strategies to improve nutrition and prevent obesity may be questionable.

## Introduction

According to the U.S. Centers for Diseases Control and Prevention (CDC), obesity prevalence in the United States (U.S.) rose from about 15% in 1994 to 27% by 2009 for all adults combined [1]. Previous epidemiological studies have demonstrated an inverse association between fruit and vegetable intake, hereafter “F&V intake”, and body weight [2,3]. In 1990, the World Health Organization (WHO) issued recommendations for a minimum daily intake of 400 g of fruits and vegetables, based on evidence that higher levels were protective to chronic diseases [4]. In 2013, WHO recommended using age-standardized mean proportion (age 18+ years) consuming less than five total servings (400 g) of fruit and vegetable per day to track effectiveness of chronic disease prevention and control [5]. Around the world, many counties and regions launched campaigns to promote and surveillance system to monitor eating five or more servings of fruits and vegetables a day, hereafter, “FV5/day”, including the U.S. [1,6–9]. Since 1989, U.S. CDC has tracked the prevalence of FV5/day among adults through the Behavioral Risk Factor Surveillance System (BRFSS) [10]. After more than 29 years of public health surveillance on the prevalence of FV5/day and obesity among U.S. adults through the BRFSS, it is important to evaluate the expected changes in FV5/day associated with chronic conditions, including obesity. Knowing whether FV5/day prevalence trends explain the increased obesity prevalence may be helpful to evaluate health disparities and inform corresponding interventions [11]. In this study, we examined whether the prevalence of FV5/day and obesity trends over time were related as expected and whether the association between FV5/day and obesity by levels of sociodemographic factors could explain the findings. The potential explanations for the evaluated relationship, especially from a methodological perspective of using FV5/day for obesity surveillance, are discussed.

## Methods

### Data

This study utilized data from the BRFSS, which is a large-scale, cross-sectional, annual telephone survey of non-institutionalized U.S. adults (≥ 18-years-old) based on random-digit dialing conducted by the individual states but coordinated by the CDC. A detailed description of the BRFSS is available elsewhere [10]. The current study focused on the nine cycles of BRFSS that included the F&V module in the rotating core questionnaire during 1994 to 2009. These years were chosen because they had all 50 states plus the District of Columbia (DC) collecting F&V intake (except for 1994 which did not have F&V data from Rhode Island) using a consistent set of survey questions. Fifty states and DC were included in the analysis. U.S. territories (Puerto Rico, Guam, and the U.S. Virgin Islands) were excluded. The number of unweighted respondents ranged from 105,853 in 1994 to 424,592 in 2009.

### Measures

F&V: Information about F&V intake derived from the six-item F&V frequency questions: “How often do you drink fruit juices such as orange, grapefruit, or tomato?”; “Not counting juice, how often do you eat fruit?”; “How often do you eat green salad?”; “How often do you eat potatoes, not including French fries, fried potatoes, or potato chips?”; “How often do you eat carrots?”; and “Not counting carrots, potatoes, or salad, how many servings of vegetables do you usually eat? For example, a serving of vegetables at both lunch and dinner would count as 2 servings”. Consistent with the national 5 A Day campaign, fried potatoes were specifically excluded. In the analysis, F&V intake among survey subjects was categorized into two groups: < 5 servings per day vs. ≥ 5 servings per day (FV5/day).

### Obesity:

Obesity was determined by body mass index (BMI) ≥ 30. BMI derived from self-reported data of weight and height and was calculated as weight (in kilograms) divided by height (in meters squared).

### Sociodemographic factors:

Sociodemographic categorizations were as follows: Age (18–39, 40–54 or ≥ 55), sex (male or female), race/ethnicity (Hispanic, white, black or other), household income (< 25 k, 25 - < 50 k, ≥ 50 k or “don’t know /refuse”), marital status (married/unmarried couple, divorced/separated, widowed or never married), and education (< high school, high school/GED, some college or technical school or 4-year college degree or more). Residence (state/DC) was grouped according to U.S. Census Bureau region (Northeast, Midwest, South or Pacific [which includes Alaska and Hawaii]). Some changes in the coding of the BRFSS variables of interest had been made over the study period; we recoded the variables to be consistent over time [10]. For this analysis, we merged race and Hispanic ethnicity together in a single variable to categorize respondents as Hispanic (of any race) and the non-Hispanics were further subcategorized as white, black, and other.

### Analysis

We estimated the yearly prevalence of FV5/day and obesity by level of sociodemographic variables (i.e., age, sex, race/ethnicity, income, education, marital status) and geographical region. We used logistic regressions to:

1. Generate yearly, predicted prevalence of FV5/day according to sociodemographic subgroups and geographical region, adjusted for other sociodemographic variables. In the multivariate logistic regression, the predicted prevalence is expressed by the regression equation. Thus, we can estimate predicted prevalence values for any combination of the values of the predictors in the regression model. In our analysis, we computed the predicted prevalence’s of the binary outcome by setting each covariate to the baseline reference value and then examined the impact of changing each one at a time.
2. Compute the prevalence odds ratio of FV5/day by obesity status and levels of sociodemographic variables and year, adjusted for other sociodemographic variables. Two-sided p-values for testing whether the odds ratios differed from 1.0 were computed. Survey-related commands (e.g. proc surveyfreq and proc survey logistic) were employed to accommodate the sampling weight and complex survey design into analyses. All statistical analyses for the study were conducted by SAS version 9.4 (SAS Institute Cary, NC).

## Results

The overall prevalence of FV5/day and obesity trends are presented in Figure 1a. The prevalence of FV5/day was stable during study period from 1994 to 2009, hovering around 25% among all U.S. adults (Figure 1a). The overall obesity prevalence increased from 14.7% to 27.4% in the same time period. During the study period, the association between obesity and FV5/day intake, as measured by predicted prevalence or odds ratios of FV5/day, remained weak. The predicted prevalence of FV5/day hovered only around 10% for the reference group (i.e. obese, male, age 18–39 years, black, < HS, < 25k income, divorced/separated, Midwest Census Region) (Figure 1b), while non-obese individuals had only 20% higher odds of having high F&V intake than obese individuals in both years (Figure 1c) but the p-values for every year were < 0.001.

A clear decreasing trend of the prevalence of FV5/day occurred in the 55+ age group. However, in comparison to younger groups, the prevalence of FV5/day remained higher throughout the years in the oldest age group (55+) (Figure 2a). The predicted prevalence of 5FV/day in age groups 55+ and 40–54 years were higher compared to the youngest age group (18–39 years) and this trend was consistent throughout the years (Figure 2b). In 1994, the oldest group was 80% more likely to have FV5/day than the youngest group. By 2009, the odds of having FV5/day in the oldest group was only around 20% higher than in the youngest group in 2009 (Figure 2c) but the p-values for every year were < 0.001. Odds of FV5/day did not differ much between age group 40–54 years and the youngest age group (but the p-values were less than or equal to 0.05 for every year except for in 2007 [p = 0.07] and 2009 [p = 0.054]).

Blacks had the lowest prevalence of FV5/day before 2002 among all racial/ethnic groups (Supplemental Figure 1a). However, they had the most increase in the prevalence of FV5/day among all race/ethnicities from 1994 to 2009 (Supplemental Figure 1a). After adjusting all sociodemographic factors, racial/ethnic disparities, as measured by odds ratio of FV5/day, narrowed over the years. For 1994 through 1998, whites, Hispanics, and other had an odds ratio significantly greater than 1.0 relative to blacks (p ≤ 0.013). For 2000 through 2009, the black-white and black-Hispanic difference regarding the odds ratio of FV5/day intake became insignificant for most years (p > 0.05) (Supplemental Figure 1b).

Large differences of the prevalence of FV5/day were observed by education group (Supplemental Figure 2a). However, the FV5/day prevalence trend in each group remained steady over time. The odds ratio of FV5/day was highest in college or above education group and lowest in high school/GED or less than high school group (Supplemental Figure 2b). Large disparities of FV5/day across education groups have lasted over the years, as the odds of FV5/day of the highest education group remained significantly higher than of the lowest education group with p-values for all years < 0.001. For people with only a high school equivalent education, the p-values are small (≤ 0.013) for 1994 through 2002, and are greater than 0.05 for the years after (Supplemental Figure 2b). Relative to age, race/ethnicity and education, smaller disparities of the odds of eating FV5/day were found in other sociodemographic subgroups, including gender, income, marital status, and geographical regions.

## Discussion

Our study revealed that the past 16 years prevalence trends of FV5/day and obesity tracked by the U.S. BRFSS are unrelated. Regardless of the levels of other adjusting variables, the prevalence of FV5/day remained low and the trend was flat from 1994 to 2009; meanwhile, the prevalence of obesity increased from 15% to 27% in the same period. This finding is conflicting with the reported inverse relationship between F&V intake and obesity [2,12].

The lack of association between F&V intake and obesity found in the study should firstly be considered in light of the methodology used for collecting the F&V data in the BRFSS. Contrary to our results, USDA’s food availability data-a national data series that is a proxy for actual food intake-showed a downward trend of F&V intake during the same time period [13]. The sensitivity of using a food frequency questionnaire to reflect actual changes in F&V intake in the U.S. is under debate [14,15,16]. Some believe this method holds certain values in measuring trends in frequency of intake over time, assuming biases in self-report remain similar [15,16]. Others argue that estimates of F&V intake from abbreviated food frequency questionnaires, such as the BRFSS module, are lower than those from other methods of dietary assessments [14]. One reason is that the BRFSS food frequency questionnaires only assess the frequency of intake (times per day) rather than servings and therefore are insensitive to changes in serving sizes. Also, food frequency questionnaires don’t include F&V intakes from mixed foods and condiments like 24-hour recalls or records; thus, it tends to underestimate the proportion of adults consuming five or more fruits and vegetables a day [17]. Additionally, like the aforementioned, food frequency questionnaires do not collect information on the preparation or physical form of F&V consumed. When the potential added energy during preparation is not taken into account, it would diminish an association between F&V intake as an intentional weight management strategy [14]. Also, F&V in mixed form is not accounted. Composite foods are frequently omitted from the response, which may lead to inaccurate estimation of F&V intake. Another limitation of BRFSS module is its lack of visual cues to illustrate portion size due to the telephone administration [18]. Additionally, the definition of F&V is not provided. For example, while tomatoes are botanically classified as fruit they are typically cooked and eaten as vegetables and therefore are counted as vegetable in the module [18]. Without proper classifications of F&V, it is likely to mislead the response. Moreover, BRFSS module used a recall timeframe of the past month or past 30 days. Even though this timeframe is common among food frequency questionnaires, it has not been rigorously evaluated whether it is ideal for accurately capturing usual intake among adults [18]. Taken together, if the estimation of FV5/day prevalence from the BRFSS deviates from its actual value, then its trend would be difficult to reflect the changes in obesity prevalence. This may serve as another underlying reason for the weak association seen in the study.

Beyond the methodological issues, another possible mechanism for the finding of unrelated prevalence trends of FV5/day and obesity maybe be due to a correlation between nutrient-dense foods and energy-dense foods. Increasing F&V intake may potentially reduce fat and energy intakes, therefore helping manage weight or facilitate weight loss. However, this would only be effective if the additional F&V displaces other higher-fat and more energy-dense foods from the diet; if not, the energy-lower benefit would be lost [19]. People may consume a diet rich in F&V but also high in calories. This may be especially the case in younger age groups who often consume larger quantities of foods that contain F&V and also energy-dense items [12]. Two earlier clinical trials suggested that when F&V intake increased without change in energy intake, weight loss did not occur [14,19]. In addition, how F&V are prepared also influences the energy density and calorie content. Unfortunately, detailed information on the preparation or physical form of F&V consumed is not collected in the BRFSS module. When F&V are fried, served with high-calorie sauces (e.g., salad dressing, butter), prepared as mixed dishes (e.g., pies, casseroles), or dried (e.g., raisins), they become energy-dense [20]. If the servings of F&V reported in the BRFSS include these less healthy forms, then the real protective effects of F&V intake on obesity are likely to be masked, thus yielding the weak association between F&V intake and obesity observed in the study.

In the evaluation of the relationship between FV5/day and obesity over time by subpopulations, we observed an expected FV5/day and obesity inverse trends over time only for older adults (≥ 55 years). Even though older adults tend to eat more F&V than their younger counterparts, less than half of them achieve the recommended servings per day [15] and they have especially low consumption of dark green and orange vegetables and legumes (12% to 15% of total vegetable consumption) [16]; instead, starchy vegetables make up a large proportion of their daily vegetable consumption [16]. Additionally, they have a significant decrease in juice consumption [17]. As shown in our data, older adults have experienced an apparent decline in FV5/day intake over time. One possible reason for the loss of F&V intake in the older group may be that their meal patterns, especially their dinner pattern, had been changing [17]. Instead of preparing and serving F&V as main or side dishes, they are opting more often for sandwich, soup, and pizza [17]. This, combined with the overall long-term trend toward simplifying the dinner meal, is contributing to their decline in FV5/day [17]. Other underlying factors for the decline in F&V intake in older adults include functional limitations, appetite changes, and dentition problems [16]. Being affected by these conditions makes consumption of F&V problematic. The lowering of FV5/day among older adults can in turn contribute to their increased obesity rate seen in the study. This finding is supported by other research that demonstrated significant health effects of F&V consumption on chronic conditions including obesity, hypertension, atherosclerosis and stroke among seniors [16,21].

There are limitations to this study. First, prevalence estimates are based on self-reports and, therefore, are subject to reporting errors. Also, as discussed previously, it is possible that estimates of F&V intake from food frequency questionnaires would not match up to actual F&V intake in the population. Additionally, although we have available information on a wide range of potential confounders, we cannot rule out uncontrolled confounding by other factors. For example, we did not include other behavioral factors that potentially interact with F&V intake and also contribute to obesity (e.g., alcohol consumption and total energy intake). However, we have adjusted the analyses for many important confounders such as sociodemographic factors and physical activities. Even after the adjustment, the unrelated association between FV5/day and obesity did not change. Lastly, we cannot quantify the contribution of fruit juice and high starchy vegetable to the FV5/day because these food items are not included in the BRFSS questionnaire. Further study is warranted to distinguish the forms and types of F&V intake and evaluate its effect on obesity.

### Implications

Lack of association between FV5/day and obesity prevalence trends over time, as measured in the BRFSS, does not mean that F&V intake should not be encouraged as a strategy for weight control. F&V intake and obesity are shown to be associated [1,2]. Fruits and vegetables characteristics of low energy density, high water content and high dietary fibers exert a risk-reducing effect on obesity, especially when they replace less healthful and high energy-dense foods [1]. If F&V intake and obesity are measured accurately, their population-level trends over time should be inverse and can be used to evaluate policy or intervention impact. For the research implications, this study highlights the need for future prospective studies, investigating the direct and independent effect of F&V intake on body weight among U.S. adults. It also stresses the need to include a more comprehensive F&V measurement, such as physical form of F&V and preparation methods in future studies to advance understanding in this field.

## Conclusion

This study evaluated the trends of FV5/day and obesity and their relationship over the study period, enabling comparison by time and by sociodemographic subgroups. Overall, the flat trend of the prevalence of FV5/day did not reflect the increasing prevalence of obesity as expected by the known relationship between F&V intake and obesity. Only in older adults was an inverse relationship between FV5/day and obesity observed. Our findings underscore the importance of improving F&V intake measurement for public health purposes and evaluating the use of FV5/day as a measure of impact of public health strategies to improve nutrition and prevent obesity in the U.S.

## Supplementary Material

1

## Figures and Tables

**Figure 1a:**
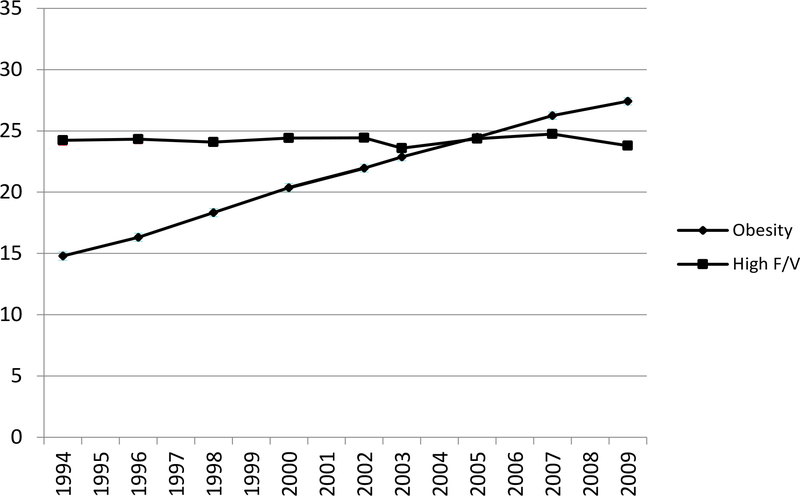
Observed overall prevalence of FV5/day and obesity prevalence in the U.S.

**Figure 1b:**
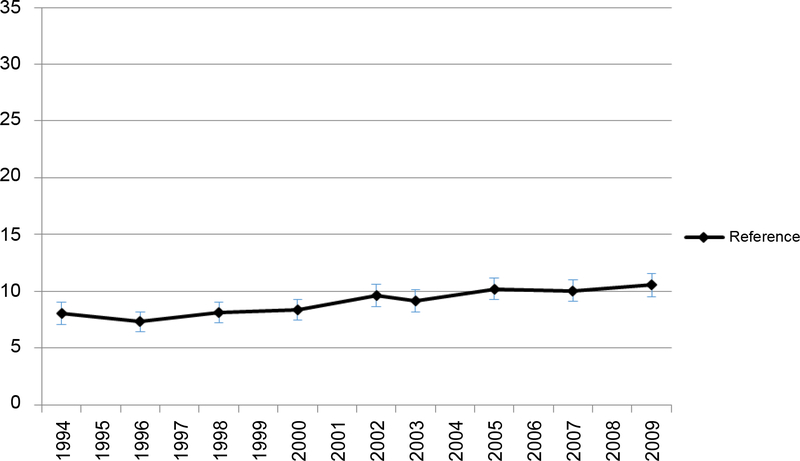
Predicted prevalence of FV5/day of the reference group (obese, male, age 18–39 years, black, < HS, obese, < 25k income, divorced/separated, Midwest Census Region).

**Figure 1c:**
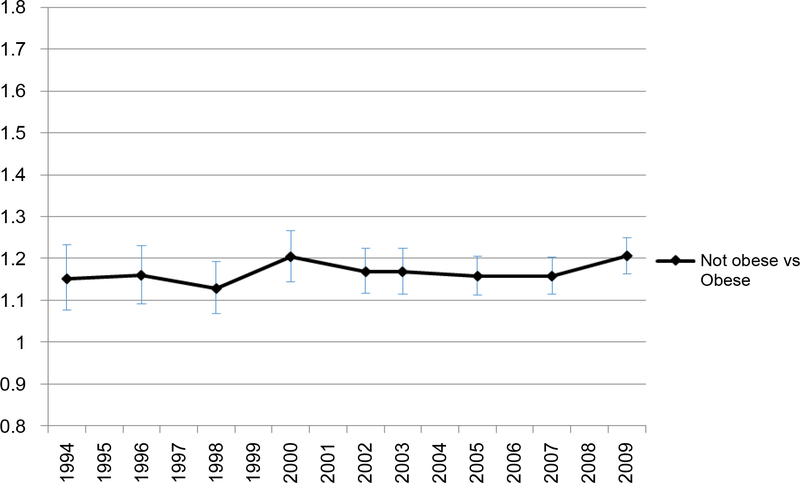
Adjusted odds ratio of FV5/day by obesity. Adjusted odds of FV5/day in non-obese group relative to odds of obese group. </p/> Covariates include age, sex, race/ethnicity, income, education, marital status, and geographical region.

**Figure 2a:**
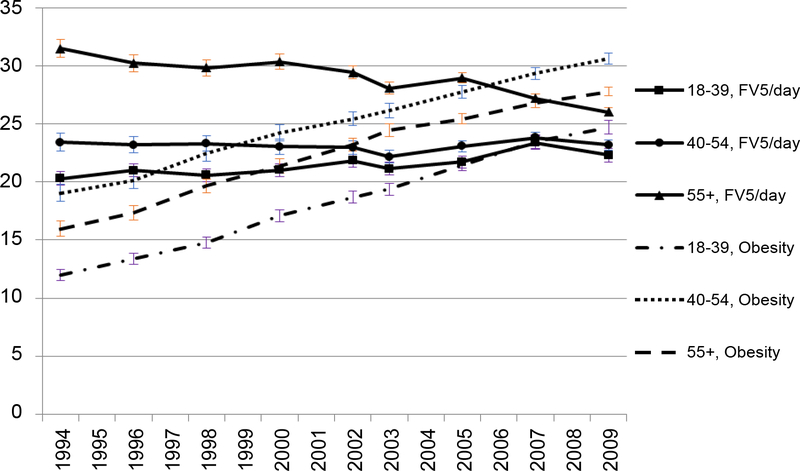
Observed prevalence of FV5/day and obesity by age.

**Figure 2b:**
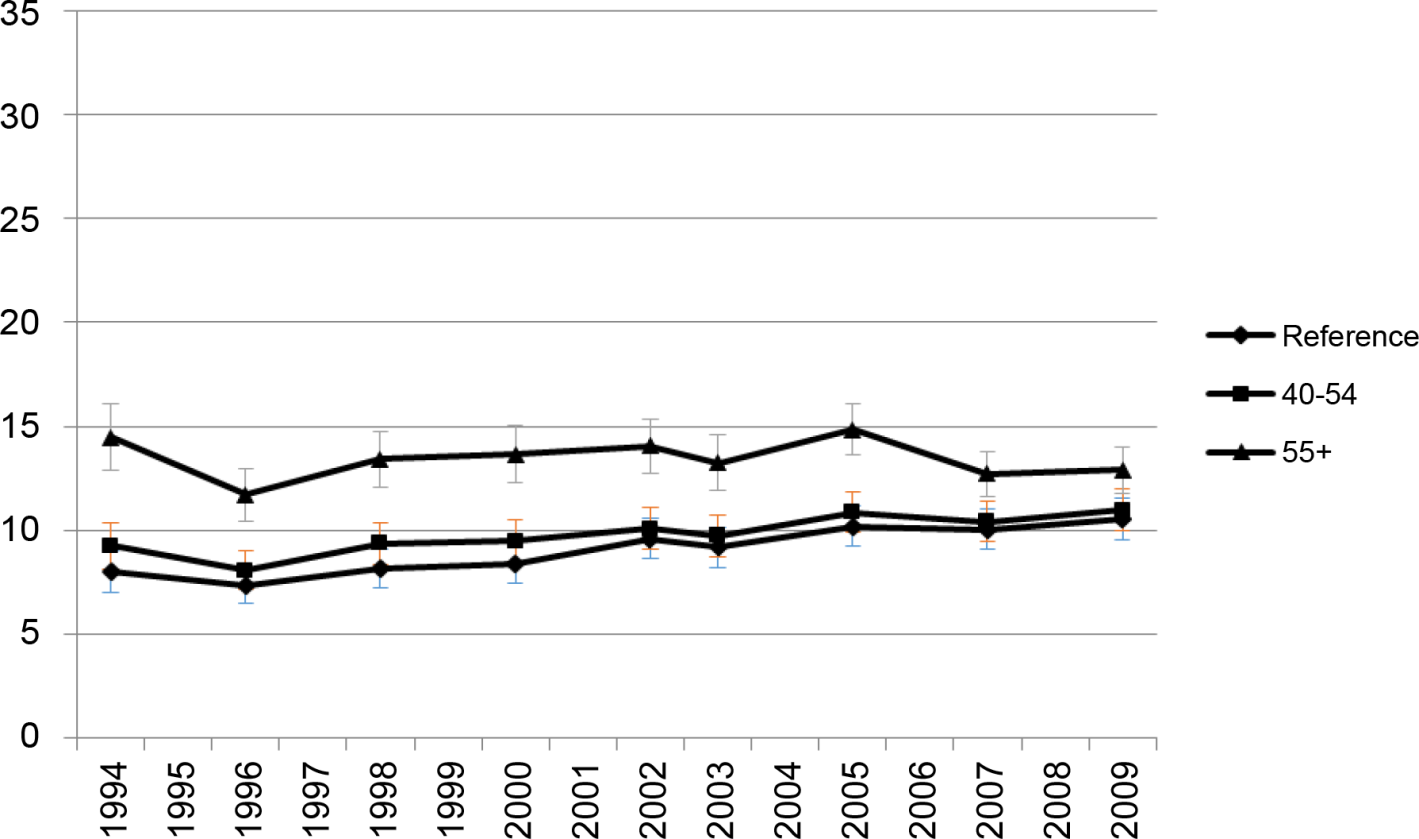
Predicted prevalence of FV5/day by age. Reference group: Age 18–39 years old.

**Figure 2c:**
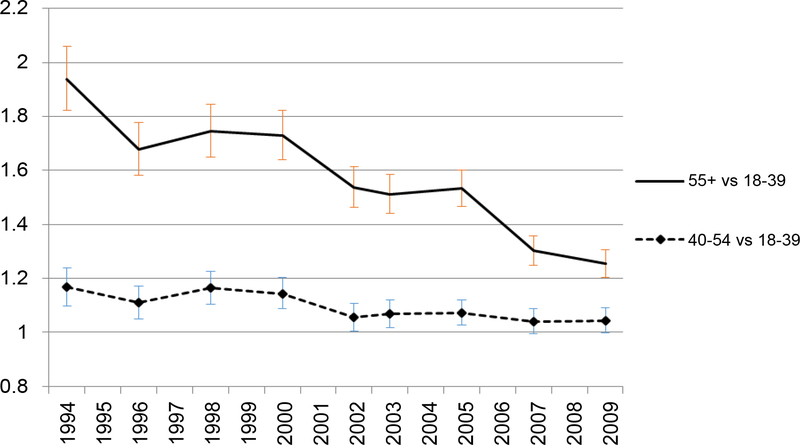
Adjusted odds ratio of FV5/day, by age. Adjusted odds of FV5/day in older age groups relative to odds in youngest group. </p/> Covariates include obesity, sex, race/ethnicity, income, education, marital status and geographical region.
