# Supplementary material for: Five Fruit and Vegetable a Day Does Not Reflect the Upward Trend of Obesity in the U.S
Source: J Nutr Med Diet Care. Author manuscript; Available in PMC 2021 Jan 18. (PMC7812612; doi:10.23937/2572-3278.1510038)
Supplement: 1 [file NIHMS1657745-supplement-1.pdf]

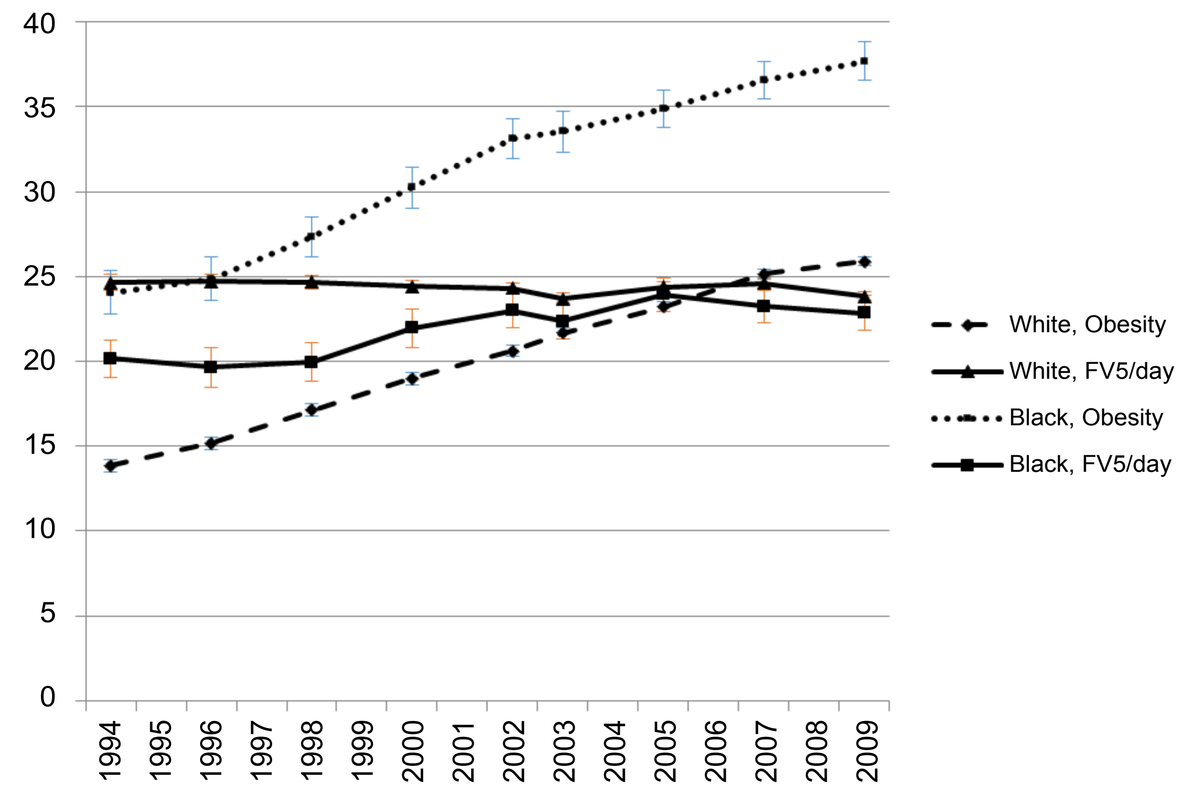

Supplemental Figure 1a: Observed prevalence of FV5/day and obesity by race/ethnicity.

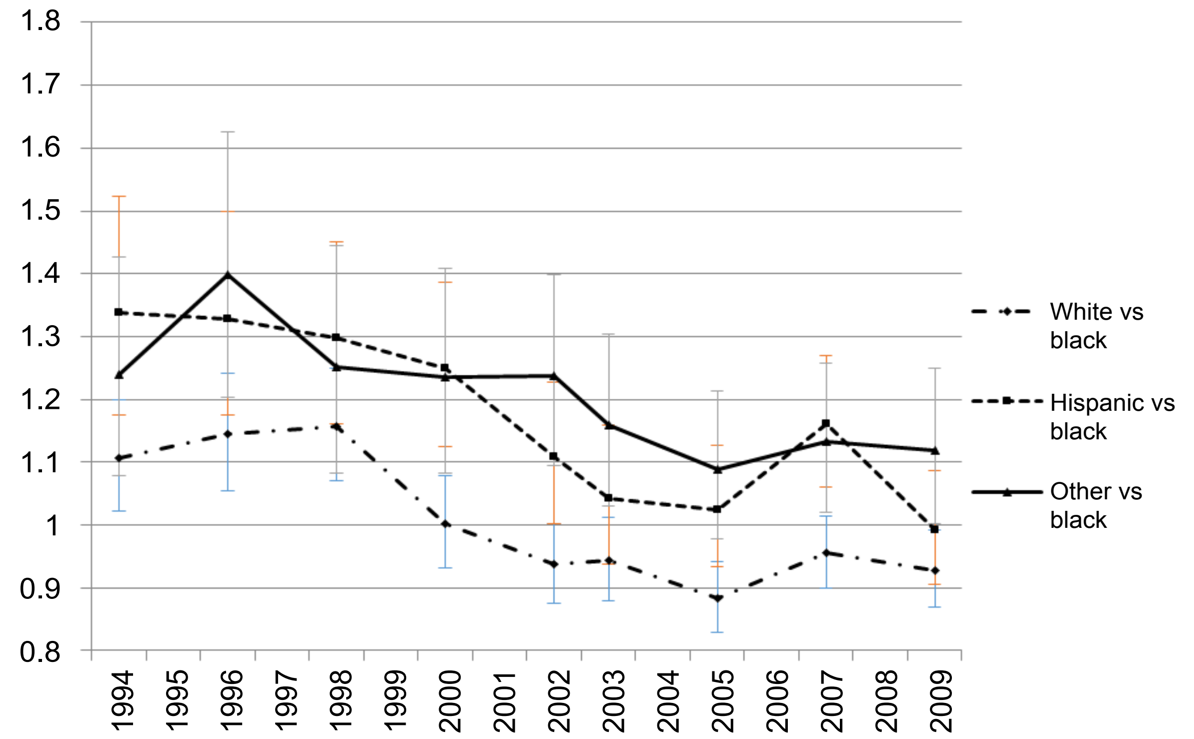

Supplemental Figure 1b: Adjusted odds ratio of FV5/day by race/ethnicity.
Adjusted odds of FV5/day in white, Hispanic and others relative to odds in black.
Covariates include obesity, age, sex, race/ethnicity, income, education marital status and geographical region.

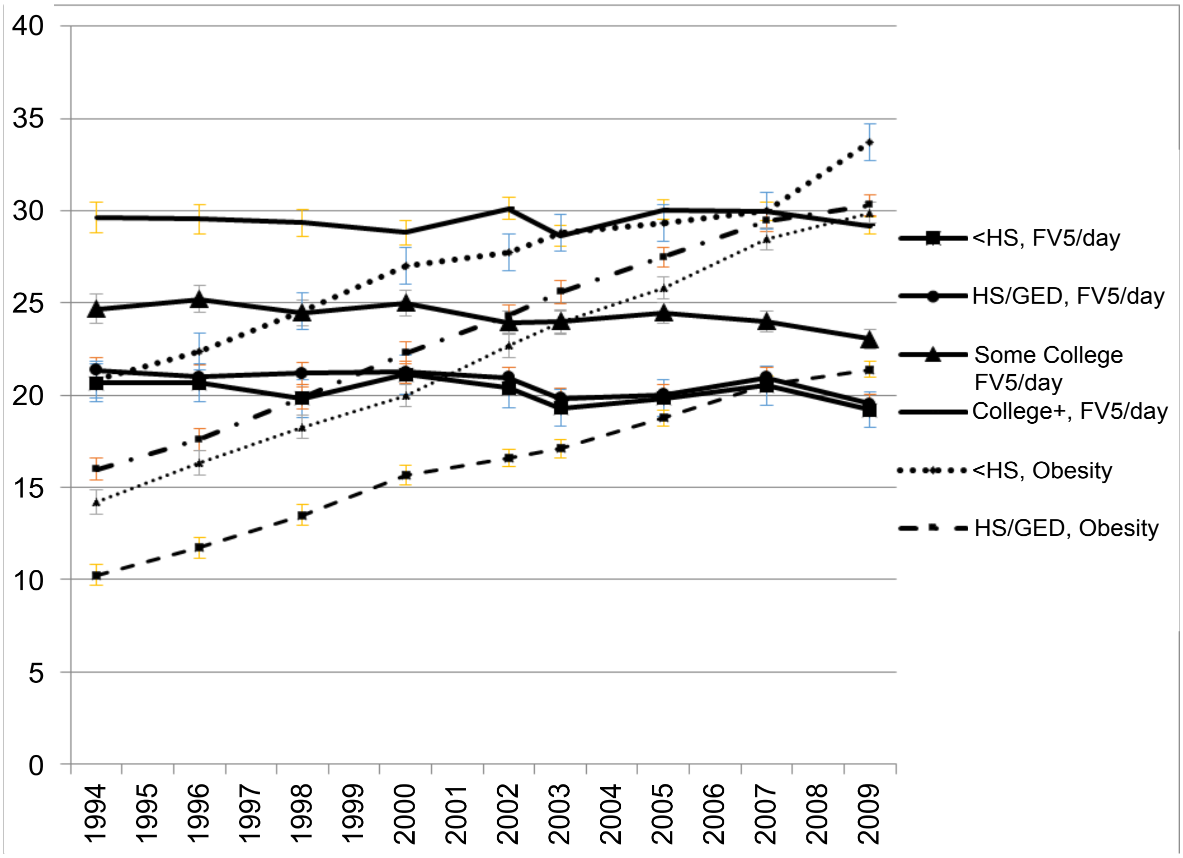

Supplemental Figure 2a: Observed prevalence of FV5/day and obesity by education.

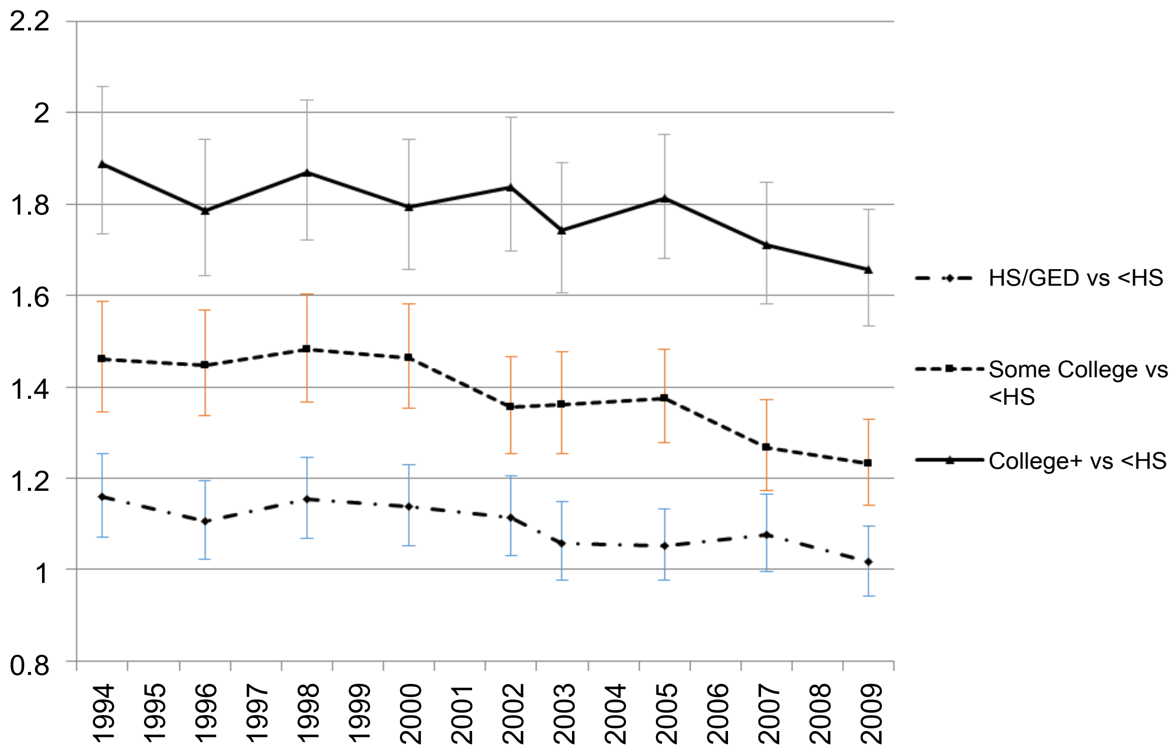

Supplemental Figure 2b: Adjusted odds ratio of FV5/day by education.
Adjusted odds of FV5/day in highest education group relative to odds in lowest education group.
Covariates include obesity, age, sex, race/ethnicity, income, marital status and geographical region.
